# Spatial and temporal patterns of vegetation carbon sources/sinks and driving factors in southeastern Xizang from 2000 to 2020

**DOI:** 10.7717/peerj.20572

**Published:** 2026-01-08

**Authors:** Jiahua Han, Xiyue Meng, Li Lin, Jie Lu

**Affiliations:** 1 School of Ecology and Environment, Xizang University, Lhasa, China; 2 Institute of Xizang Plateau Ecology, Xizang Agricultural and Animal Husbandry University, Nyingchi, China; 3 Key Laboratory of Forest Ecology in Xizang Plateau, Xizang Agricultural and Animal Husbandry University, Ministry of Education, Nyingchi, China; 4 National Forest Ecosystem Observation and Research Station of Nyingchi Xizang, Nyingchi, China

**Keywords:** Net ecosystem productivity, Southeast Xizang (Tibet), CASA model, GeoDetector, Driving factors

## Abstract

Net ecosystem productivity (NEP) is a crucial measurement for understanding ecosystem function and carbon cycling. On the basis of Moderate Resolution Imaging Spectroradiometer (MODIS) data, meteorological data, and topographic data, combined with the enhanced Carnegie Ames Stanford Approach (CASA) model, we analyzed the spatiotemporal trends and changes in the vegetation NEP in southeastern Xizang from 2000 to 2020. Additionally, we employed the GeoDetector model to identify the driving factors influencing the vegetation NEP. The results indicated that: (1) from 2000 to 2020, the annual vegetation NEP in southeastern Xizang exhibited a fluctuating increasing trend. The multiyear average vegetation NEP was 519.06 gC m^−2^ a^−1^, ranging from 11.23 to 1,333.40 gC m^−2^ a^−1^. The minimum and maximum values occurred in 2010 and 2015, respectively. (2) The spatial distribution pattern of the vegetation NEP revealed an overall trend of higher values in southern areas and lower values in northern areas, with higher values in eastern areas compared to western areas. The average area of the carbon source regions (NEP < 0) was approximately 70,119 km^2^, whereas the average area of the carbon sink regions (NEP > 0) was approximately 82,017 km^2^. Overall, the region exhibited a carbon sink characteristic. (3) Altitude, precipitation, and temperature were the primary drivers influencing the vegetation NEP. In regions at lower elevations and in the southern and eastern parts of the study area—where thermal and moisture conditions are relatively favorable—NEP values were generally higher. In contrast, NEP was markedly lower in the northern and high-elevation areas characterized by low temperatures and limited water availability. The interactions between any two of these factors had a greater impact on the vegetation NEP than the independent effect of any single factor did, highlighting a synergistic dual-factor enhancement effect.

## Introduction

Since the Industrial Revolution, the advent of the steam engine, the development of industrial mechanization, and the large-scale combustion of fossil fuels such as coal, oil, and natural gas for energy production, transportation, and industrial manufacturing have led to a significant increase in atmospheric CO_2_ concentrations (Lu et al., 2023). The rising concentration of atmospheric CO_2_ has led to various climate and environmental problems, including droughts, floods, sea level rise, warming, unpredictable weather, and changes in rainfall patterns (Moomaw, Law & Goetz, 2020; Pepin, 2021). Global surface temperatures are continuously rising (IPCC, 2007). Global warming has had a significant impact on the carbon cycle (Luo et al., 2009; Zhou et al., 2019). Research on the carbon cycle has garnered considerable attention from governmental bodies and the scientific community alike. In recent years, numerous nations have implemented a suite of responsive measures aimed at substantially curbing greenhouse gas emissions, enhancing global climate stability, air quality, water resources, and ecosystem health, and fostering the sustainable advancement of the global socioeconomic landscape (Jia et al., 2022; Zhang et al., 2023a). China has established clear emission reduction targets: striving to achieve a peak in CO_2_ emissions before 2030 and to reach carbon neutrality before 2060 (Friedlingstein et al., 2020; Zhang et al., 2023a). In addition, China’s Nationally Determined Contribution (NDC) outlines a series of specific targets, including reducing CO_2_ emissions per unit of GDP by more than 65% from the 2005 level by 2030, increasing the share of non-fossil fuels in primary energy consumption to around 25%, expanding forest stock volume by approximately 6 billion m^3^ compared with 2005, and raising the total installed capacity of wind and solar power to over 1,200 GW. These measures reflect China’s concrete actions toward optimizing its energy structure, enhancing forest carbon sinks, and accelerating the expansion of renewable energy (Lin & Peng, 2025; Zuñiga, Forner & Bill, 2025). Terrestrial ecosystems are essential to the global carbon cycle, serving not only as indicators of regional environmental conditions but also revealing the impacts of human activities on ecosystems (Bonan, Lombardozzi & Wieder, 2021; Friend et al., 2014).

Currently, research on the carbon cycle in terrestrial vegetation has progressed from estimating gross primary productivity (GPP) and net primary productivity (NPP) to evaluating net ecosystem productivity (NEP), determining regional carbon balances, identifying carbon sources and sinks, and quantifying the effects of environmental changes and anthropogenic influences on vegetation carbon stocks. This includes a detailed quantitative evaluation of how environmental changes and anthropogenic influences affect vegetation carbon reserves (Francesca Cotrufo et al., 2021; Huang, Chen & Zhou, 2020). GPP represents the total amount of carbon fixed by vegetation through photosynthesis and serves as the “entry point” of the ecosystem carbon cycle, reflecting the overall potential of vegetation to absorb atmospheric CO_2_. NPP is the portion of GPP remaining after subtracting autotrophic respiration (Ra), expressed as NPP = GPP − Ra, and represents the accumulation of organic carbon available for plant growth and energy transfer within the food chain. NEP is the difference between NPP and heterotrophic respiration (RH). It measures the net carbon uptake or storage in vegetated ecosystems, providing a quantitative assessment of their capacity to act as carbon sources or sinks (Keenan et al., 2016; Li et al., 2020). RH refers to the CO_2_ released during the decomposition of organic matter by soil microorganisms and fauna, which is distinct from autotrophic respiration by vegetation and constitutes an important component of soil respiration. Its magnitude is typically regulated by factors such as temperature, precipitation, and soil physicochemical properties, and can be estimated at the regional scale using empirical equations (*e.g.*, temperature–precipitation models) or process-based models. If NEP > 0, the ecosystem acts as a carbon sink, whereas if NEP < 0, it acts as a carbon source (Qiu et al., 2023). Thus, as an important parameter characterising vegetation activity, accurate estimation of NEP is useful for assessing the health of vegetation ecosystems.

Numerous studies have focused on the net ecosystem productivity (NEP) of terrestrial ecosystems. predominantly concentrating on assessing its effects, identifying its characteristics, and elucidating the underlying mechanisms of change. In terms of assessing NEP effects, extensive research has been conducted globally and across various countries and regions, with a primary focus on estimating the average annual NEP. Some scholars have focused on studying the average annual NEP per unit area of terrestrial ecosystems. Yamaji et al. (2008) integrated Moderate Resolution Imaging Spectroradiometer (MODIS) data with flux-based observations to generate NEP maps for Japanese deciduous forests spanning 2002 to 2003, employing scaling-up techniques. Yu et al. (2013) studied the carbon flux dynamics across various terrestrial ecosystems in China, highlighting that these regions are significantly influenced by annual mean temperature and precipitation. Zhang et al. (2019) studied the influence of climate factors on the interannual NEP variability, highlighting precipitation as the primary driver of NEP fluctuations in China. He et al. (2018) used models to estimate NPP and NEP in the Yellow River. Precipitation was found to be the primary limiting factor, and diurnal asymmetric warming correlated significantly with NEP, particularly the impact of nighttime temperatures on vegetation cover. Guo et al. (2021) employed the Carnegie Ames Stanford Approach (CASA) model and a geostatistical soil respiration model to estimate the NEP in the Hindu Kush Himalaya (HKH) region from 2001 to 2018, revealing spatial and temporal variations in carbon sources/sinks, with grasslands showing potential carbon sequestration capacity influenced by elevation and precipitation patterns. Zhang et al. (2021) revealing spatiotemporal variations in the NEP in the Central Asia ecosystem from 2001 to 2019 and highlighting the significant impact of climate change, particularly temperature and precipitation, on carbon flux dynamics. Existing studies have shown that climatic factors, particularly temperature and precipitation, are key drivers of the spatiotemporal variations in NEP and determine whether terrestrial ecosystems act as carbon sinks or sources at different stages. Although research on vegetation carbon source–sink dynamics on the Tibetan Plateau remains relatively limited, some scholars have conducted exploratory studies (Xu, Guo & Zhang, 2024; Geng et al., 2024), providing valuable references for further investigation into the spatiotemporal evolution and driving mechanisms of carbon sources and sinks in this region.

The Qinghai-Xizang Plateau is the highest plateau in China (3,000–5,000 m) (Shi, Lu & Yu, 2024; Yang et al., 2023). The southeastern region of Xizang is one of the best preserved areas of primeval forests in China. The forest vegetation is diverse and includes tropical, subtropical, temperate and humid climates. Typical endemic tree species include *Cupressus gigantea*, *Abies georgei* var. *smithii*, *Alsophila spinulosa*. The ecosystem productivity of this region is relatively low, its regulatory capacity is comparatively weak, and it stands out as one of the most climate-sensitive areas in China. Therefore, it is of paramount importance to conduct studies on the NEP of ecosystems and its influencing factors in this region.

In recent years, numerous studies have analyzed vegetation carbon sinks and their driving mechanisms across the Tibetan Plateau, identifying the dominant environmental factors at the plateau scale and covering a wide range of regions (Dong et al., 2024). This study used high-resolution remote sensing data, an improved Carnegie Ames Stanford Approach (CASA) model, and meteorological and topographical data, to study the spatial and temporal changes in the NEP in the region from 2000 to 2020, and explore the differences in the distributions of carbon sources/sinks and the drivers of the NEP in the region. The research results not only contribute to a deeper understanding of the carbon balance dynamics in southeastern Xizang but also provide a scientific basis for the effective management and enhancement of regional carbon storage. Moreover, they offer important insights for formulating carbon cycle regulation and ecological conservation strategies tailored to plateau regions.

## Study Area and Data

### Study area

Nyingchi city is located in the southeastern part of the Xizang Autonomous Region and is situated between 26°52′ and 30°40′N latitude and 92°09′ and 98°47′E longitude. It borders India and Myanmar, with elevations ranging from 150 to 7,782 m above sea level, exhibiting significant topographical variation. It is located at the confluence of the Nyang River and Yarlung Tsangpo River, nestled to the north by the Nyainqentanglha Range, and bordered to the south by the Himalayas, with remnants of the Gangdise Mountains to the northwest and the Hengduan Mountains to the east. The climate of Nyingchi city is predominantly a maritime monsoon climate, which is also influenced by the alpine climate and the Xizang Plateau monsoon climate. Summers are relatively warm and humid, whereas winters are relatively cold and dry. The average temperature ranges from 6 to 17 °C, which is higher than that in other regions at similar latitudes. The annual and diurnal temperature variations are relatively small. The region receives abundant rainfall, with precipitation concentrated mainly in the summer months from June to September, exhibiting irregular distribution patterns. The annual precipitation in most parts of Nyingchi city ranges from 500 to 1,000 mm (Yongxiang et al., 2022). However, due to topographical influences, significant variations exist between different areas: the western Yarlung Tsangpo River basin receives less than 500 mm annually, whereas southern parts of the Himalayas, such as Motuo County, may receive more than 2,000 mm of rainfall annually.

Nyingchi city boasts a rich diversity of vegetation types, including evergreen broadleaf forests, evergreen coniferous forests, mixed conifer-broadleaf forests, shrublands, grasslands, and permanent snowfields. Evergreen broadleaf forests primarily inhabit valleys at altitudes ranging from 1,500 to 3,000 m. Evergreen coniferous forests and mixed conifer–broadleaf forests, featuring various species, such as *Abies fabri*, *Picea asperata*, and *Larix gmelinii*, extend between 3,000 and 4,000 m. Shrublands and grasslands dominate the high plateau regions, which are typically found at altitudes ranging from 3,500 to 5,000 m. Additionally, mountain summits exceeding 5,000 m are blanketed with extensive permanent snowfields, serving as crucial water sources for numerous rivers (Shi, Lu & Yu, 2024; Yang et al., 2023).

### Data collection and processing

(1) Meteorological data were obtained from TerraClimate (available at https://climate.northwestknowledge.net/TERRACLIMATE/index_animations.php). TerraClimate is a gridded dataset containing monthly climate and climate water balance data for global land surfaces from 1958 to 2022. This dataset is essential for ecological and hydrological studies requiring high spatial and temporal resolution, with data available at a spatial resolution of 4 kilometers (approximately 1/24th of a degree) and a temporal resolution of one month (Abatzoglou et al., 2018).

(2) Vegetation type data are derived from the global 30 m fine ground cover dynamic monitoring product (GLC_FCS30–1985_2020) (Zhang et al., 2023c), which was released by Liu Liangyun’s team for the period 1985–2020. This product contains 29 ground cover types. The update cycle is five years.

(3) The Normalized Difference Vegetation Index (NDVI data) is primarily used to indicate vegetation cover, leaf area index, vegetation growth vigor, and ecosystem productivity, serving as an important indicator for studying vegetation dynamics and the carbon cycle. NDVI data from Landsat-7 are provided by the USGS (https://www.usgs.gov/landsat-missions/landsat-collection-2-surface-reflectance). The NDVI formula is as follows: NDVI = (NIR-Red)/(NIR+Red), where “NIR” represents the near-infrared band of the Landsat-7 satellite, and “Red” represents the red band (after atmospheric correction).

(4) The MOD17A3H data, sourced from NASA, originally has a spatial resolution of 500 m and a temporal resolution of one year. To ensure consistency in spatial resolution, the data was resampled to 30 m for validating the NPP values computed by the model.

### Data quality control and methodological robustness

#### Data quality control and SLC-OFF gap handling

To address the SLC-OFF striping gaps and cloud/shadow interference in Landsat-7 imagery after 2003, a rigorous data quality control procedure was applied. Pixels affected by clouds, cloud shadows, snow/ice, and saturation were first removed using the QA flags from the Collection 2 Surface Reflectance (SR) dataset, with a cloud probability threshold of 20%. Within the growing season (June–September), multiple images acquired in the same month were screened for quality to derive monthly NDVI composites, and an annual medoid composite was then generated to represent each year, allowing the temporal synthesis process to naturally fill the striping gaps. Pixels with fewer than three valid observations were flagged for subsequent filtering. To reduce systematic biases caused by observation geometry and terrain effects, Bidirectional Reflectance Distribution Function (BRDF) normalization was performed using MCD43 parameters, combined with terrain correction based on the SCS+C model and SRTM DEM.

#### Spatial and temporal alignment of multi-source data

A unified spatial resolution of 30 m × 30 m was adopted as the target grid. The spatial resolutions of Landsat-7 NDVI and GLC_FCS30 (30 m) were already consistent with the target grid, and land-cover data were mapped by category without interpolation. TerraClimate variables, including monthly mean temperature, total precipitation, and total solar radiation (4 km), were resampled to 30 m using bilinear interpolation and co-registered with NDVI and land-cover grids to drive the CASA model on a monthly scale. MOD17A3H annual NPP (500 m) was used as an external reference and resampled to 30 m for pixel-to-pixel comparison. Temporally, all datasets were aligned on a monthly basis: monthly NDVI was used to estimate the fraction of photosynthetically active radiation (FPAR) and *ɛ*, which, together with monthly meteorological data, were used to calculate monthly NPP; soil heterotrophic respiration was also simulated monthly and subsequently accumulated to obtain annual NEP.

#### Discretization of continuous factors and robustness testing

For the analysis of driving factors, continuous variables such as precipitation, temperature, elevation, and slope were discretized into five classes using the natural breaks (Jenks) method to balance within-group variance minimization and sample size uniformity, while aspect, as a categorical variable, was used directly. To assess the robustness of results, both equal-interval and equal-frequency classifications were applied for comparison. Although the absolute q-statistics differed slightly across classification schemes, the relative ranking and interaction types of dominant factors remained consistent, indicating robust conclusions.

### Methods

#### NPP estimation

The CASA model, proposed by Potter et al. (1993), is a typical large-area-scale light energy use model driven by a combination of remotely sensed data, meteorological data, and land cover data. The NPP depends on how much photosynthetically active radiation (APAR) it absorbs relative to its light energy conversion efficiency (*ɛ*). Zhu, Pan & Zhang (2007) enhanced the existing CASA model by incorporating vegetation cover classification into the model. The NPP formula for the improved CASA model is as follows:

(1) \begin{eqnarray*}{\mathrm{NPP}}_{\mathrm{(x,t)}}={\mathrm{APAR}}_{\mathrm{(x,t)}}\times {}_{\mathrm{(x,t)}}\end{eqnarray*}

where NPP is the net primary productivity of vegetation, $\mathrm{APA}{\mathrm{R}}_{ \left( \mathrm{x},\mathrm{t} \right) }$ is the photosynthetically active radiation absorbed by image x in month t (MJ m^−2^), and ${\epsilon }_{ \left( \mathrm{x},\mathrm{t} \right) }$ is the efficiency of light energy utilization by image x in time period t.

The absorbed photosynthetically active radiation (APAR) in a specific area is determined by the proportion of total incoming radiation that the vegetation absorbs, calculated using the following formula:

(2) \begin{eqnarray*}{\mathrm{APAR}}_{\mathrm{(x,t)}}={\mathrm{SOL}}_{\mathrm{(x,t)}}\times {\mathrm{FPAR}}_{\mathrm{(x,t)}}\times 0.5\end{eqnarray*}

where SOL_(x,t)_ is the total solar radiation (MJ m^2^) received within pixel x in the t-th time period, FPAR_(x,t)_ is the fraction of photosynthetically active radiation absorbed by the vegetation within pixel x in the t-th time period, and 0.5 denotes the proportion of total solar radiation used for photosynthesis.

The total solar radiation was computed using an established formula developed by He and Xie (He and Xie., 2010), which is specific to the development of western China:

(3) \begin{eqnarray*}\mathrm{Q}={\mathrm{Q}}_{0}\times (\mathrm{a}+\mathrm{b}\times {\mathrm{T}}_{\mathrm{S}}/{\mathrm{T}}_{\mathrm{A}})\end{eqnarray*}

where Q_0_ is astronomical radiation, which is calculated on the basis of geographic latitude, solar declination and other parameters; T_S_ is observed sunshine hours; T_A_ is theoretical sunshine hours, which is calculated on the basis of latitude and solar declination; and a and b are empirical coefficients (*a* = 0.185; *b* = 0.595).

The formula for calculating the fraction of photosynthetically active radiation absorbed (FPAR) by vegetation is as follows:

(4) \begin{eqnarray*}{\mathrm{FPAR}}_{\mathrm{(x,t)}}=\alpha {\mathrm{FPAR}}_{\mathrm{NDVI}}+(1-\alpha ){\mathrm{FPAR}}_{\mathrm{SR}}\end{eqnarray*}

(5) \begin{eqnarray*}FPA{R}_{NDVI}& = \frac{NDV{I}_{(x,t)}-NDV{I}_{(i,\min \nolimits )}}{NDV{I}_{(i,\max \nolimits )}-NDV{I}_{(i,\min \nolimits )}} \times (FPA{R}_{\max \nolimits }-FPA{R}_{\min \nolimits })+FPA{R}_{\min \nolimits }\end{eqnarray*}

(6) \begin{eqnarray*}FPA{R}_{SR}& = \frac{S{R}_{(x,t)}-S{R}_{(i,\min \nolimits )}}{S{R}_{(i,\max \nolimits )}-S{R}_{(i,\min \nolimits )}} \times (FPA{R}_{\max \nolimits }-FPA{R}_{\min \nolimits })+FPA{R}_{\min \nolimits }\end{eqnarray*}

(7) \begin{eqnarray*}S{R}_{(x,t)}& = \frac{1+NDV{I}_{(x,t)}}{1-NDV{I}_{(x,t)}} \end{eqnarray*}

where t is the month and the rest of the parameters are as above. *FPAR*_max_ and *FPAR*_min_ are the maximum and minimum photosynthetically active radiation ratios taken as 0.95 and 0.001, respectively. *SR*_(*i*,max)_ and *SR*_(*i*,min)_ are the maximum and minimum values of the simple ratio of the normalized index for the ith vegetation type, obtained in a probability distribution of 95% and 5%, respectively. *NDVI*_(*i*,max)_ and *NDVI*_(*i*,min)_ are the maximum and minimum values of the NDVI for the ith vegetation type, taking the DN values corresponding to a probability distribution of 95% and 5%. *SR*_(*x*,*t*)_ is the simple ratio of the normalized vegetation index within pixel x in time period t.

The actual light use efficiency (*ɛ*) is influenced primarily by temperature, water availability, and vegetation type. The effective light use efficiency is determined by multiplying the vegetation’s maximum light use efficiency by factors related to water and temperature stress. The calculation formula is as follows:

(8) \begin{eqnarray*}{}_{\mathrm{(x,t)}}={T}_{1(\mathrm{x,t})}\times {T}_{2(\mathrm{x,t})}\times {W}_{(\mathrm{x,t)})}\times {}_{\mathrm{max}}\end{eqnarray*}

where T_*ɛ*1_ and T_*ɛ*2_ are the stress effect coefficients of the maximum and minimum temperatures on the actual light use efficiency *ɛ*_(x,t)_, W_*ɛ*(x,t)_ is the water stress effect coefficient, and *ɛ*_max_ is the maximum light energy utilization under ideal conditions (gC MJ^−1^).

Table 1 lists the NDVI_(i,min)_, NDVI_(i,max)_, NDVI_(i,min),_ NDVI_(i,max)_ and *ɛ*_max_ values of the different vegetation types (Zhu, Pan & Zhang, 2007).

**Table 1:** NDVI_(i,min)_, NDVI_(i,max)_, NDVI_(i,min),_ NDVI_(i,max)_ and *ɛ*_max_ values of different vegetation types.

Vegetation type	NDVI_max_	NDVI_min_	SR_max_	SR_min_	*ɛ*_max_
Evergreen needleleaved forest	0.647	0.023	4.67	1.05	0.389
Deciduous coniferous forest	0.738	0.023	6.63	1.05	0.485
Deciduous broadleaf forest	0.747	0.023	6.91	1.05	0.692
Mixed forest	0.702	0.023	5.84	1.05	0.475
Shrubland	0.636	0.023	4.49	1.05	0.429
Grassland	0.634	0.023	4.46	1.05	0.542
Cropland	0.634	0.023	4.46	1.05	0.542
Water	0.634	0.023	4.46	1.05	0.542
Impervious surface	0.634	0.023	4.46	1.05	0.542
Barren	0.634	0.023	4.46	1.05	0.542

#### NEP estimation

NEP serves as a critical indicator of regional vegetation carbon dynamics, representing the difference between NPP and carbon lost through soil microbial respiration, excluding other natural and human-induced influences, which is calculated as follows:

(9) \begin{eqnarray*}\mathrm{NEP(x,t)}=\mathrm{NPP(x,t)}-\mathrm{RH(x,t)}\end{eqnarray*}

where *NEP*(*x*, *t*) represents the net ecosystem productivity of vegetation for pixel x during time period t (gC m^−2^), *NPP*(*x*, *t*) represents the net primary productivity of vegetation for pixel x during time period t (gC m^−2^), and *RH*(*x*, *t*) represents the soil microbial respiration of pixel x during time period t (gC m^−2^).

The calculation formula for soil heterotrophic respiration is as follows (Zhiyong et al., 2003):

(10) \begin{eqnarray*}{\mathrm{R}}_{\mathrm{H}}=3.069\times ({\mathrm{e}}^{0.0912\mathrm{T}}+\mathrm{In}(0.3145\times \mathrm{R}+1))\end{eqnarray*}

where R_H_ represents soil heterotrophic respiration (gC m^−2^), T represents the temperature (°C), and R represents precipitation (mm).

#### Theil–Sen trend analysis and testing methods

The Theil–Sen median (Sen) method is less affected by outliers, making it more robust. (Lu et al., 2023). It is a widely used metric for analyzing trends in long-term data series. It measures the direction and magnitude of trends in NEP: a positive β signifies an upward trend, while a negative β signifies a downward trend. The formula for calculating β is as follows:

(11) \begin{eqnarray*}\mathrm{\beta }=\mathrm{Median}({\mathrm{NEP}}_{\mathrm{j}}-{\mathrm{NEP}}_{\mathrm{i}})/(\mathrm{j}-\mathrm{i}) 2000\leq i< j\leq 2020\end{eqnarray*}

where b is Sen’s slope value, and NEP_i_ andNEP_j_ are the NEPs in years i and j, respectively. The Mann−Kendall test was used to test the significance of the results of the β-trend analyses.

#### GeoDetector

The GeoDetector model is a tool for detecting spatial heterogeneity and is capable of revealing the drivers behind spatial differentiation (Zhang et al., 2023b); it includes four modules: the factor detector, interaction detector, risk detector, and ecological detector. This study employed the GeoDetector model to investigate the driving factors of spatial variation in the NEP of vegetation in southeastern Xizang.

Factor detection: this tool assesses the extent to which the driving factor X (Average multiyear total precipitation (2000–2020, mm); Average multiyear average temperature (2000–2020, ^∘^C); Digital Elevation Model (DEM, m); Slope (^∘^); Aspect (^∘^) (°C)) influences Y (NEP), employing the *q* value metric to quantify this impact. which is calculated as follows:

(12) \begin{eqnarray*}q=1- \frac{1}{f{\sigma }^{2}} \sum _{h=1}^{L}fk{\sigma }^{2}k\end{eqnarray*}

where L is the classification of factor X or variable Y; *f* and *fk* represent the sample sizes in the entire study area and within type h, respectively; and *σ*^2^ and *σ*^2^*k* represent the discrete variances of Y values across the entire study area and within type h, respectively. The value range of q is [0, 1]. A higher *q* value signifies that the driver factor X has a stronger explanatory power on the NEP, while a lower *q* value indicates a weaker explanatory power.

Interaction detector (GeoDetector software 1.6, developed by Professor Jiumiao Cheng’s team at Renmin University of China; freely available at http://geodetector.cn/index.html): a statistical tool used to evaluate the impact of multiple factors on a specific variable. By detecting the interactions between different factors, the interaction detector can reveal how these factors work together and whether their combined effects are synergistic, antagonistic, or independent. Details of the interaction types are given in Table 2.

**Table 2:** Types of driver interactions.

Interaction type	Interactive relationship
Nonlinear attenuation	q(X_1_∩X_2_) <Min[q(X_1_),q(X_2_)]
Single-factor nonlinear attenuation	Min[q(X_1_),q(X_2_)] <q(X_1_∩X_2_) <Max[q(X_1_),q(X_2_)]
Dual-factor enhancement	q(X_1_∩X_2_) >Max[q(X_1_),q(X_2_)]
Independent	q(X_1_∩X_2_)= q(X_1_)+q(X_2_)
Nonlinear enhancement	q(X_1_∩X_2_) >q(X_1_)+q(X_2_)

### Model accuracy validation

The MOD17A3 vegetation NPP data product was selected to validate the CASA model estimates, the MOD17A3 data (500 m resolution) were resampled to the same spatial resolution scale as the data in this study (30 m resolution), 100 sample points within the study area were randomly selected, a total of 500 sample points over five years were used, and sample point data of MOD17A3 NPP values and CASA model estimated NPP values were used to test the correlation between the two. The correlations were tested using a linear regression model, with R^2^ used to evaluate the goodness of fit, and statistical significance was assessed by Pearson correlation analysis (*p* < 0.01). All analyses and plotting were conducted in Origin 2021.

## Results and Analyses

### Accuracy validation

**Figure 1:**
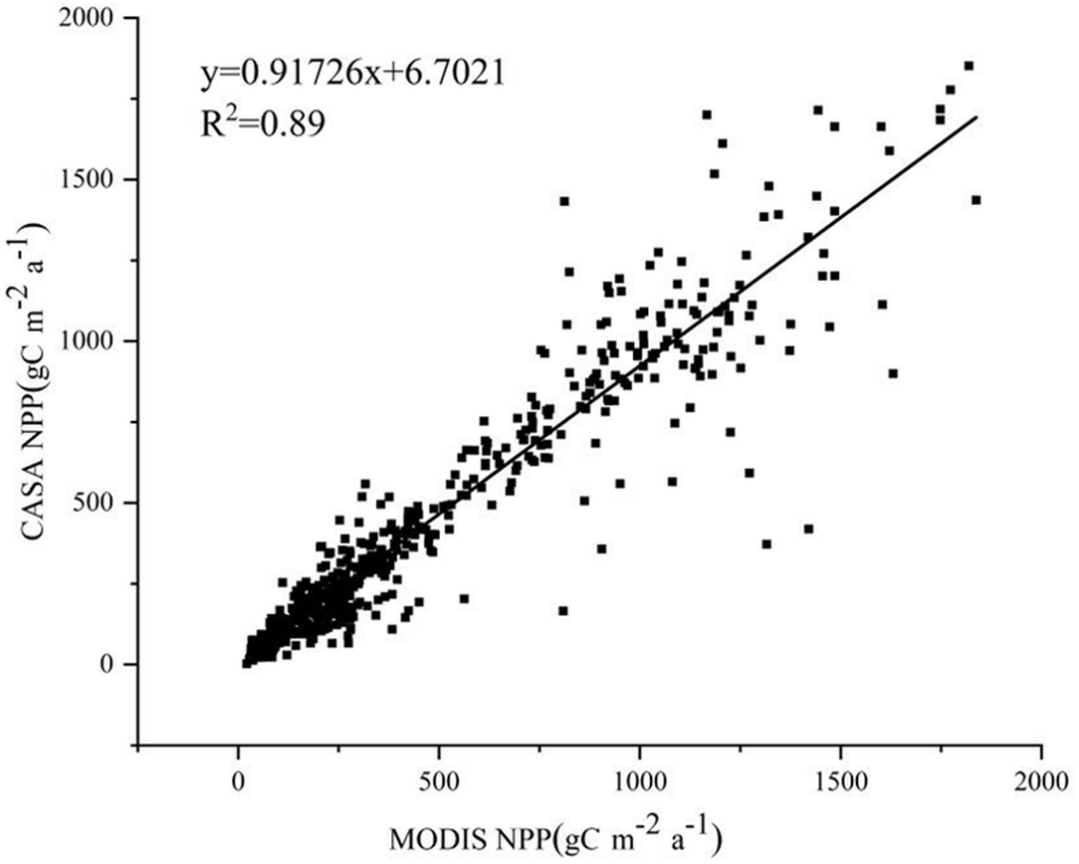
Accuracy of NPP estimation *via* the CASA model.

In this study, the CASA model was used to estimate NPP data at a spatial resolution of 30 m. The MOD17A3 data product was used as a reference to validate the estimation results. The NPP values estimated by the CASA model show a good fit with those from the MOD17A3 data product, with a linear regression equation of *y* = 0.91726x+6.7021 and an R-squared value of 0.89. There was good consistency between the two products (Fig. 1).

### Temporal variation characteristics of the NEP

**Figure 2:**
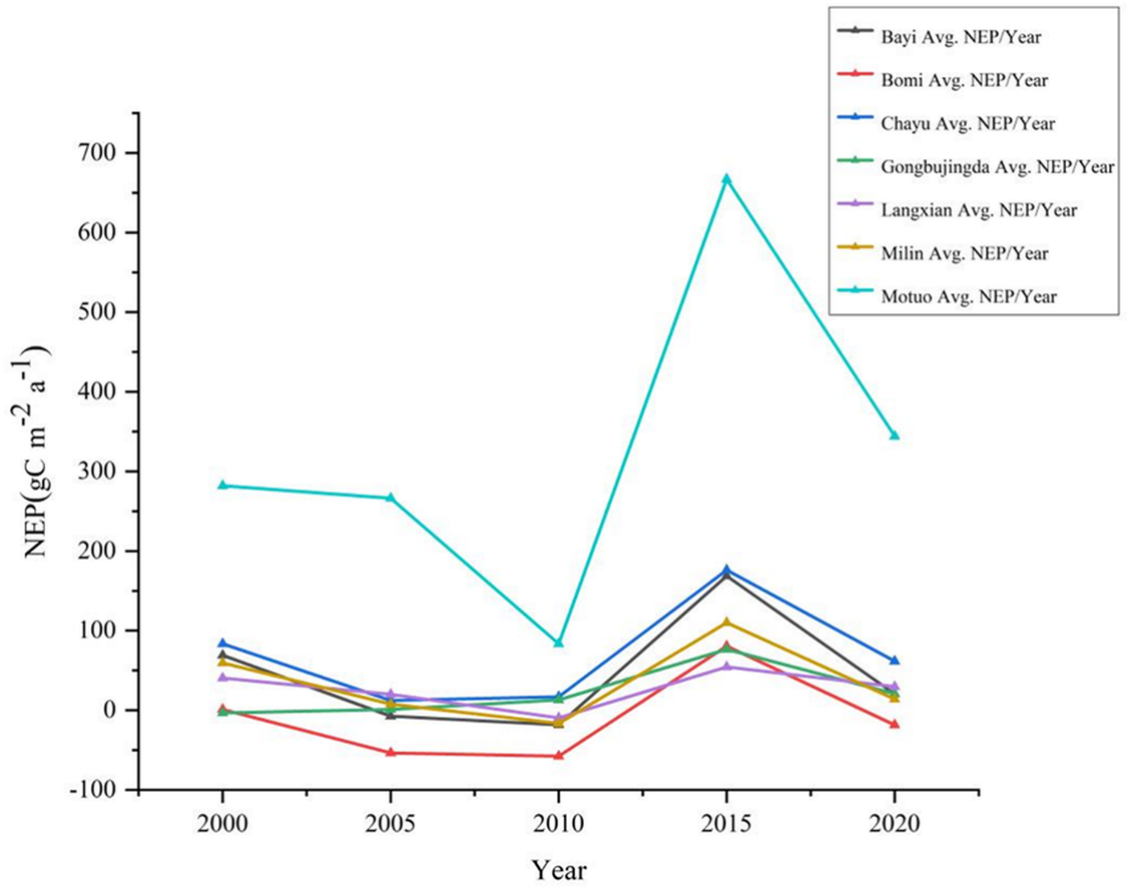
Changes in the total annual NEP in different regions of southeastern Xizang, 2000–2020. Net ecosystem productivity (NEP) < 0 carbon sink, Net ecosystem productivity (NEP) > 0 carbon source.

From 2000 to 2020, the vegetation NEP in southeastern Xizang exhibited a fluctuating but overall increasing trend (Fig. 2). The multiyear average value of the vegetation NEP was 519.06 gC m^−2^ a^−1^, ranging from 11.23 to 1333.40 gC m^−2^ a^−1^. The minimum and maximum values occurred in 2010 and 2015, respectively, and the largest fluctuation during these five years was 117.74 gC m^−2^ a^−1^. Among the different regions in southeastern Xizang, Motuo County presented the highest multiyear average NEP value of 328.55 gC m^−2^ a^−1^, whereas Bomi County presented the lowest multiyear average NEP value of only −9.63 gC m^−2^ a^−1^, indicating a carbon source status. During the period from 2000 to 2010, the NEP in southeastern Xizang exhibited a decreasing trend. Compared with the period from 2015 to 2020, the study area had relatively lower vegetation cover, characterized by a climate of drought and low precipitation, which is unfavorable for vegetation growth. Consequently, the overall carbon sequestration capacity of the vegetation was relatively low. With the climate gradually warming and becoming more humid, vegetation growth conditions have improved, extending the growing season and leading to an overall increase in photosynthetic carbon fixation by vegetation.

Overall, the multiyear NEP of vegetation in southeastern Xizang showed a fluctuating upward trend from 2000 to 2020, indicating a carbon sink status for the region. Vegetation carbon sequestration exceeded soil respiration, and the carbon sequestration capacity of vegetation increased continuously. This improvement can be partly attributed to various ecological conservation measures implemented in southeastern Xizang, such as the establishment of nature reserves, the implementation of rotational grazing bans, afforestation projects, and soil and water conservation initiatives. These efforts have helped to improve conditions for vegetation growth.

### Spatial variation characteristics of the NEP

**Figure 3:**
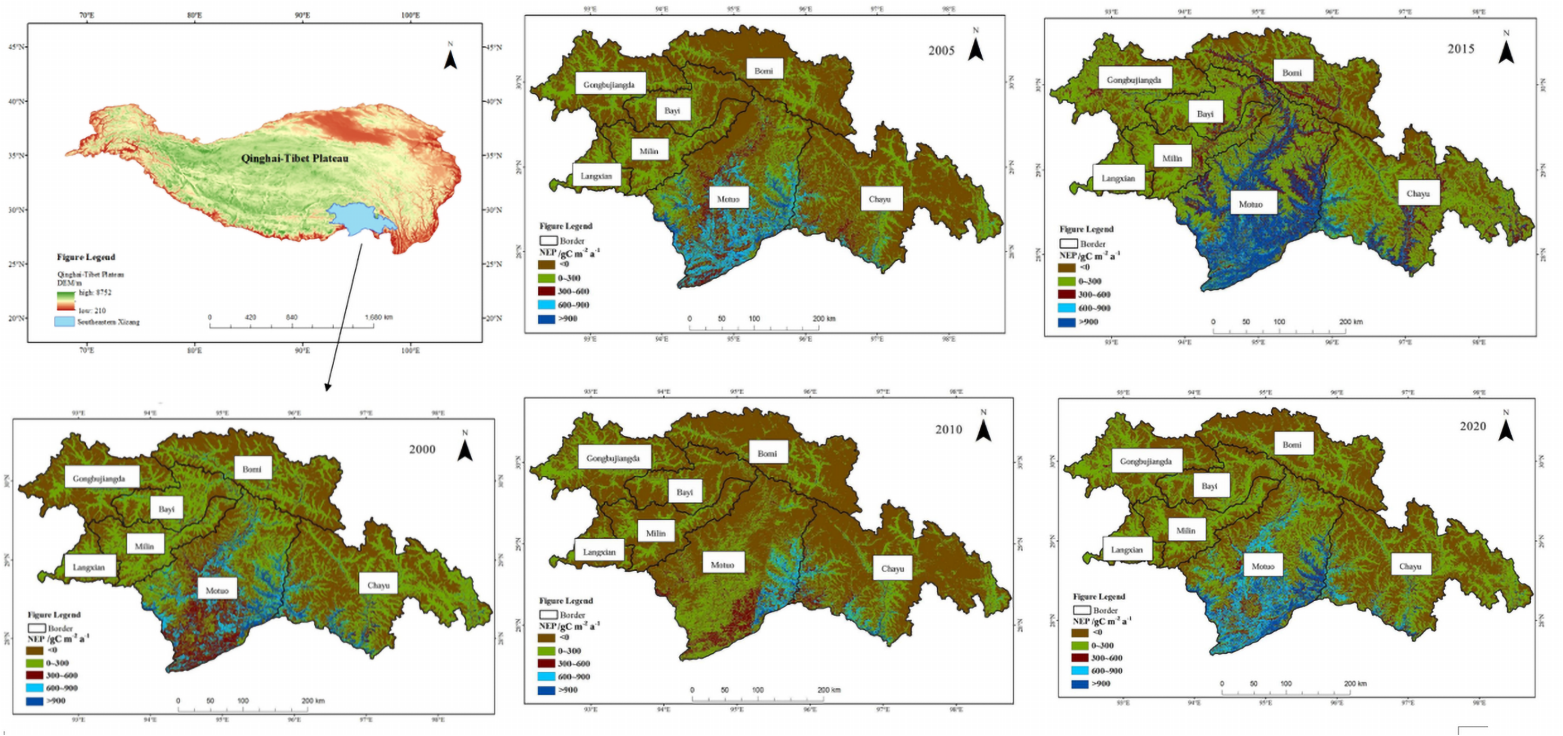
Spatial variation in NEP in southeastern Xizang, 2000–2020. Net ecosystem productivity (NEP) < 0 carbon sink, Net ecosystem productivity (NEP) > 0 carbon source.

The spatial distribution pattern of NEP in Southeast Xizang from 2000 to 2020 shows a trend of higher values in the south and lower values in the north, as well as higher values in the east and lower values in the west (Fig. 3). The annual mean total vegetation NEP in southeastern Xizang was 519.06 gC m^−2^ a^−1^, with approximately 70 119 km^2^ classified as a carbon source area (NEP < 0) and approximately 82 017 km^2^ as a carbon sink area (NEP > 0), indicating a predominantly carbon sink characteristic. The average area of vegetation with NEP values of 0–300 gC m^−2^ a^−1^ in the entire study area was approximately 57,194 km^2^. The areas with NEP values of 300–600 gC m^−2^ a^−1^ covered an average of approximately 11,007 km^2^, whereas those with values of 600–900 gC m^−2^ a^−1^ covered approximately 6,481 km^2^. Vegetation areas with NEP values exceeding 900 gC m^−2^ a^−1^ averaged approximately 5,668 km^2^. Areas with NEP values 0–300 gC m^−2^ a^−1^ was most widespread within the study area.

The high-value areas of vegetation NEP in southeastern Xizang were located primarily in Motuo County and Chayu County. These regions experience a subtropical mountain monsoon climate, with average temperatures of 20.1 °C and 11.9 °C and annual precipitation of 2,198.3 mm and 807.8 mm, respectively. The vegetation in Motuo and Chayu Counties is dominated by primary forests comprising various vegetation types, such as tropical rainforest, subtropical evergreen deciduous forest, and mixed coniferous and deciduous forest. The vegetation exhibits lush growth and possesses a strong carbon sequestration capacity. Motuo County has annual vegetation NEP values ranging from 83.44 to 667.01 gC m^−2^ a^−1^, whereas Chayu County has annual vegetation NEP values ranging from 12.39 to 176.19 gC m^−2^ a^−1^. In southeastern Xizang, Bomi County has the lowest vegetation NEP, making it the only carbon source area in the region. From 2000 to 2020, the average vegetation NEP in this area was −9.62 gC m^−2^ a^−1^, Bomi County experiences a plateau temperate monsoon climate, with an average annual temperature of 8.7 °C and an annual rainfall of 901.5 mm. The vegetation mainly consists of alpine coniferous forests and alpine deciduous forests. Compared with other regions in southeastern Xizang, the vegetation in this area has a lower carbon sequestration capacity. The multiyear average vegetation NEP values in Gongbujiangda County, Langxian, Milin County, and Bayi District were 21.56 gC m^−2^ a^−1^, 26.99 gC m^−2^ a^−1^, 34.92 gC m^−2^ a^−1^ and46.54 gC m^−2^ a^−1^, respectively.

### Analysis of the NEP trend in southeastern Xizang

**Figure 4:**
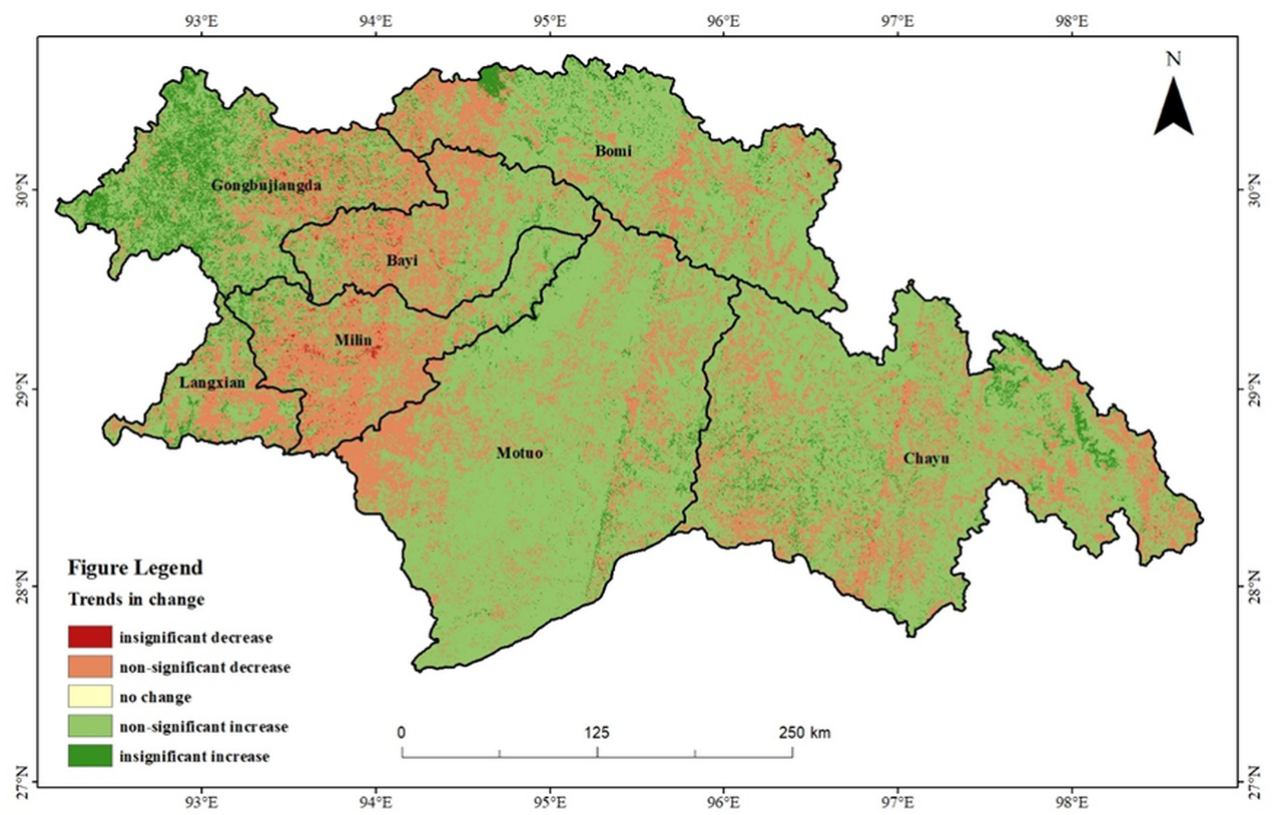
Vegetation NEP trends in southeastern Xizang, 2000–2020.

To gain a clearer understanding of the spatial and temporal variability in vegetation NEP within the study area, Sen’s trend analysis and the Mann–Kendall test were applied for an image-by-image meta-analysis. The operations were first performed in Matlab R2022b and then further processed in ArcMap 10.8, and classified into five categories: non-significant decrease, insignificant decrease, no change, insignificant increase and non-significant increase. The trend map of the vegetation NEP in southeastern Xizang from 2000 to 2020 revealed that most areas exhibited a non-significant increasing trend (Fig. 4). The area with a multiyear increasing trend in vegetation NEP covered 108,542 km^2^, accounting for 72.74% of the entire study area. This indicated generally good vegetation growth in southeastern Xizang, which was primarily distributed in Motuo County, the western part of Gongbujiangda County, and parts of Chayu County, Bomi County, and Bayi District. The area with a multiyear decreasing trend in the vegetation NEP covered 41,925 km^2^, accounting for approximately 27.86% of the total area. This trend was primarily distributed in most parts of Milin County and Bayi District, with scattered occurrences in other regions.

### Driving factors of the NEP in southeastern Xizang

**Figure 5:**
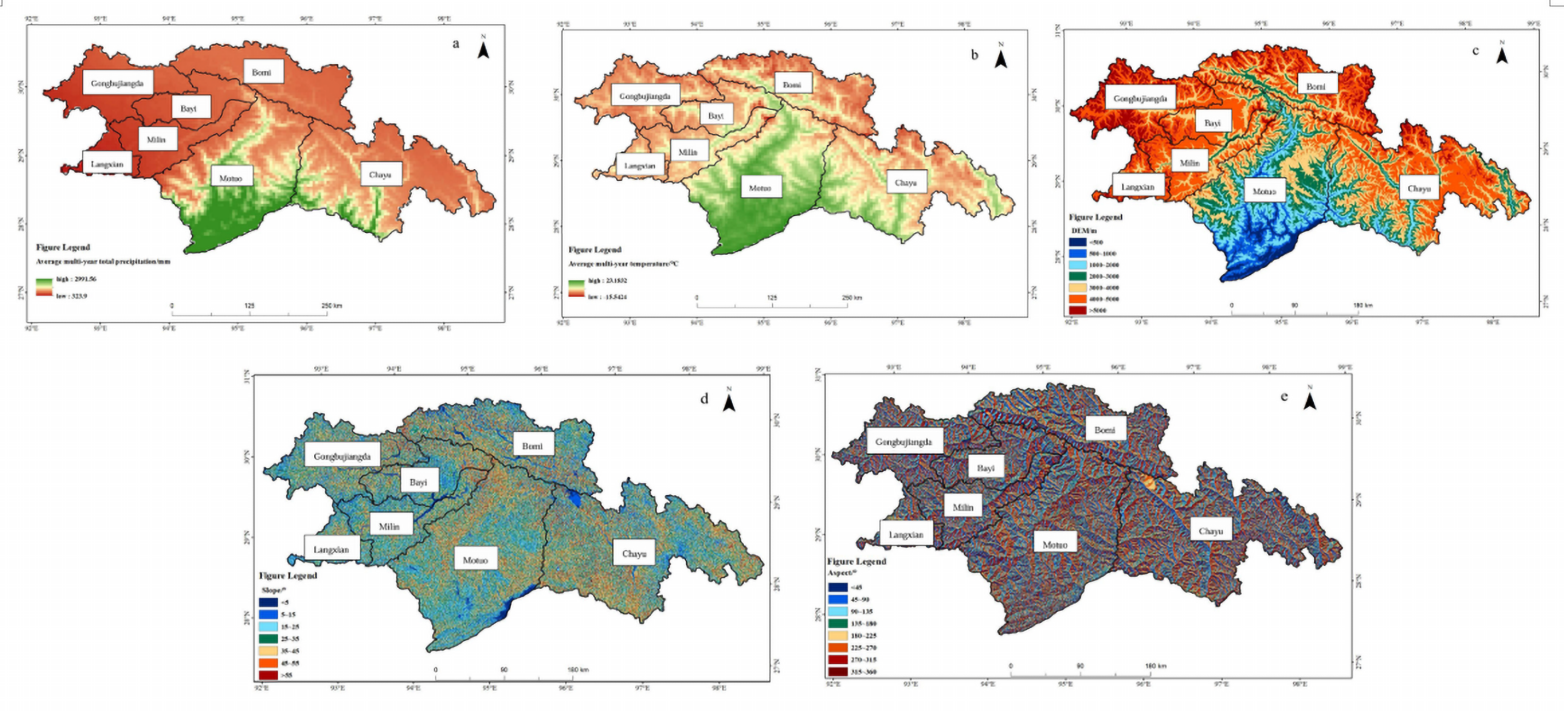
Spatial distribution of natural drivers in southeastern Xizang. (A) Average multi-year total precipitation (2000–2020, mm; here “total” refers to the annual total precipitation averaged over 2000–2020, rather than the sum of all years); (B) Average multi-year temperature (2000–2020, °C; here “average” refers to the annual mean temperature averaged over 2000–2020); (C) Digital Elevation Model (DEM, m); (D) Slope (°); (E) Aspect (°).

Vegetation carbon sequestration is influenced by multiple factors, which can be broadly categorized into three types: climatic factors, such as precipitation, temperature, and solar radiation; other natural factors, including topography, vegetation types, and soil properties; and human activities related to land planning measures. In this study, five natural factors, namely, average multiyear total precipitation (Fig. 5A), average multiyear average temperature (Fig. 5B), altitude (Fig. 5C), slope (Fig. 5D), and aspect (Fig. 5E), were selected to analyze their effects on the NEP of vegetation in southeastern Xizang. ArcGIS software was used to geoprocess each factor to obtain its spatial distribution (Fig. 5), which was then analyzed *via* GeoDetector 1.6 software.

**Figure 6:**
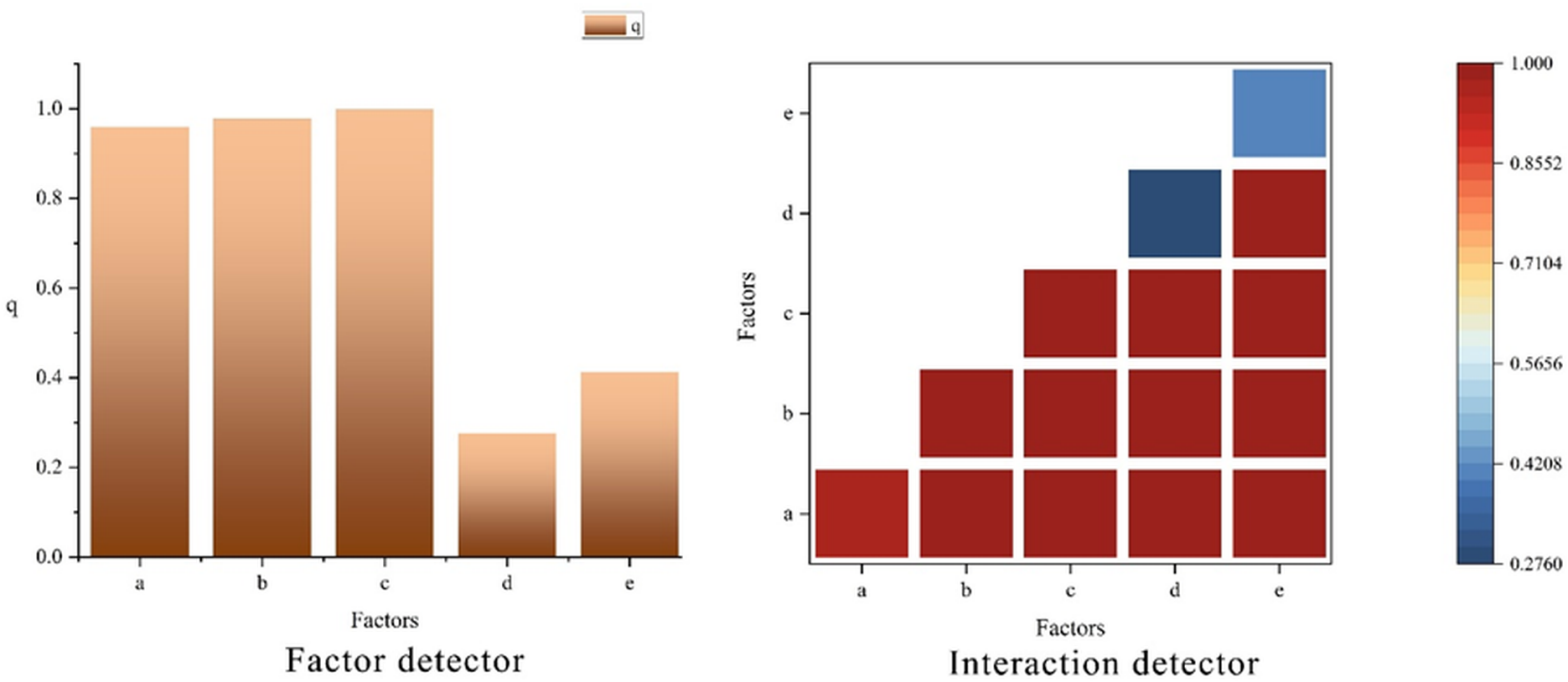
Factor detection results for NEP in southeastern Xizang. (A) Average multiyear total precipitation (2000–2020, mm); (B) Average multiyear average temperature(2000–2020, °C); (C) Digital Elevation Model (DEM, m); (D) Slope (°); (E) Aspect (°) The factor detector bar chart quantifies the explanatory power of each driving factor for the spatial heterogeneity of NEP; a larger q statistic indicates a stronger explanatory effect. The interaction detector heat map depicts the explanatory power of pairwise interactions between factors, with the color scale representing the interaction q value (0–1); higher values denote stronger joint explanatory effects.

The factor detection results (Fig. 6, factor detector) revealed that altitude had the strongest explanatory power for the spatial distribution of the vegetation NEP in the study area, followed by the average multiyear average temperature and average multiyear total precipitation. Aspect exhibited relatively weak explanatory power, whereas slope had the lowest explanatory power for the vegetation NEP in the study area. Altitude, precipitation, and temperature were identified as key factors that caused spatial heterogeneity in the vegetation habitats. Spatially, the driving factors exhibited a distinct spatial differentiation pattern in explaining the vegetation NEP in the study area. Precipitation, temperature, and altitude were the primary factors influencing the vegetation NEP in the study area.

When the interaction detector was used to analyze the interactions between different driving factors and their impacts on vegetation NEP spatial heterogeneity (Fig. 6, interaction detector), the interaction effects of any two driving factors on vegetation NEP were greater than the independent effects of individual factors alone, and all pairwise combinations appeared as red color blocks in the figure, indicating synergistic enhancement due to the interaction between factors. This suggests that there is a synergistic interaction among the driving factors, and the spatial-temporal variation in the vegetation NEP in southeastern Xizang cannot be determined by a single driving factor but rather by the combined influence of multiple factors. Precipitation, temperature, and altitude had the strongest explanatory power when interacting with other driving factors, highlighting them as the most important drivers influencing the vegetation NEP in the study area. In contrast, factors such as slope and aspect showed relatively weaker explanatory power.

## Discussion

### Uncertainty in NEP estimates

Before discussing the spatial and temporal variations and driving factors of NEP, it is essential to ensure the reliability of the model results. Therefore, this section first evaluates the accuracy and uncertainty of NEP estimates based on the CASA model.

Assessing the accuracy of vegetation NEP estimates at the regional scale has been a difficult and highly controversial aspect of remote sensing estimation. The CASA model is a large-scale, or even global, NPP estimation model based on the entire vegetation of North America. In recent years, scientists have greatly improved the accuracy of the model by modifying its parameters, considering its regional adaptability, and improving the resolution of remote sensing data (Liang et al., 2015; Liu et al., 2018; Qi, Zhang & Zhang, 2023). There are usually two commonly used methods to validate the accuracy of estimation models: The first method involves validating the accuracy of estimated values by comparing them with actual measurements; the second is the relative method, which involves comparing the estimated spatial distribution maps with other remote sensing data products to assess their accuracy. Although measured data from field surveys are more reliable, conducting large and uniform field survey samples across the study area is challenging, due to the significant spatial and temporal heterogeneity of biomass distributions, which can introduce errors. In this paper, the accuracy of the CASA model estimates was verified *via* the MOD17A3 vegetation NPP data product. The results revealed that the spatial distribution of the NEP is similar to the findings of Chuai et al. (2018) and Zhang, Zhao & Wu (2023). The vegetation NEP trend analysis results are similar to those of Lu et al. (2023). Therefore, it can be assumed that the NEP estimation results in this paper have high credibility.

Quality control and scale harmonization measures were applied during data processing; however, these methods may also introduce certain uncertainties. The annual NDVI compositing process may smooth extreme values, potentially underestimating vegetation peaks in some areas. Downscaling meteorological data from 4 km to 30 m may obscure fine-scale variations, and resampling MOD17A3H NPP data to 30 m for comparison could lead to sub-pixel mixing effects. For GeoDetector, the discretization of continuous factors can influence the magnitude of q values, but the ranking and interaction patterns of key factors remained consistent across different classification schemes, indicating that the overall conclusions are robust.

### Spatial and temporal changes in the NEP in southeastern Xizang

During the study period, the multiyear vegetation NEP in southeastern Xizang showed a fluctuating upward trend, indicating that the region overall acted as a carbon sink. Spatially, the vegetation NEP exhibited a pattern of being greater in the southern and eastern regions and lower in the northern and western regions. These findings are consistent with the results of Lu et al. (2023) and Sun et al. (2015).

The minimum and maximum values of the vegetation NEP in the study area occurred in 2010 and 2015, respectively, with the largest fluctuations during these five years being 117.74 gC m^−^^2^ a^−^^1^. This trend was influenced by various factors, with vegetation growth being regulated primarily by climatic factors (Richardson et al., 2013). The vegetation NEP values are closely related to the amount of solar radiation and water the plants receive. In 2010, the climate was relatively dry with little rainfall. From 2010 to 2015, the vegetation NEP increased significantly. The acceleration of global climate warming and humidification, along with various ecological protection measures implemented in southeastern Xizang (Liu, Li & Lai, 2021; Tomar et al., 2023), created favorable conditions for vegetation growth.

There were clear spatial distribution characteristics, with Motuo County having the highest vegetation NEP. This was the result of the combined effects of climatic conditions and vegetation richness. Huang et al. (2023) reported that the higher the altitude is, the poorer the soil moisture and nutrient conditions, and consequently, the poorer the vegetation biomass and productivity. Motuo County, with its relatively low altitude and abundant precipitation, has ample water and heat, making it a high-value area for the vegetation NEP in southeastern Xizang. In contrast, Gongbujiangda County, Langxian, and Milin County in the northwest, with their higher altitudes and stronger ultraviolet radiation, faced climatic limitations in terms of vegetation growth. These factors resulted in low spatial distributions of NEP values.

### Analysis of drivers of the NEP in southeastern Xizang

The carbon cycle is an important cycle of matter in ecosystems (Zeng et al., 2023), and maintaining the ecological balance is paramount. Significant natural variations across different regions of southeastern Xizang result in varying vegetation types and distribution ranges. As a result, the characteristics of the vegetation NEP vary under different natural conditions. For southeastern Xizang, precipitation, temperature and altitude were the most important drivers affecting the vegetation NEP in the study area. An interaction detector was used to analyze the interactions between different drivers and their effects on the spatial differentiation of the vegetation NEP, which revealed that the interaction of any two drivers had a greater effect on the vegetation NEP than the independent effect of a single factor did, indicating a two-factor amplification effect.

Temperature and humidity are the main meteorological variables affecting vegetation growth (Samadianfard et al., 2019). Studies have confirmed the substantial impact of temperature and precipitation on NEP changes across various spatial and temporal scales (Christopher Oishia et al., 2019; Wang et al., 2021b). Temperature and precipitation not only directly affect vegetation growth but also indirectly regulate the NEP through their effects on R_H_. Suitable temperatures and precipitation tend to promote photosynthesis and vegetation growth and increase carbon fixation capacity (Chen & Zhang, 2023). Elevated temperatures tend to accelerate microbial metabolic activities and increase the decomposition rate of soil organic matter, thereby increasing R_H_ values (Santana, Gonzalez & De Recursos, 2015; Woo & Seo, 2021). Moreover, precipitation is also critical in regulating soil moisture. Suitable soil moisture can promote microbial activity and organic matter decomposition, but too much precipitation can lead to soil water saturation and inhibit oxygen diffusion, thus inhibiting R_H_ (Santana, Gonzalez & De Recursos, 2015). This study revealed a significant effect of topography (altitude, slope, slope aspect) on the vegetation NEP in the study area, which is similar to the findings of several other researchers (Chen et al., 2024; Guo, 2023; Wang et al., 2021a; Yuan et al., 2021). With increasing elevation, ecosystems face multiple constraints driven by both climatic and topographic factors. In particular, air temperature decreases markedly and the growing season shortens, which has been shown to be a key factor limiting GPP and net carbon uptake in alpine tundra ecosystems of the European Alps (Magnani et al., 2020).In ecosystems above 3,000 m in the Nepal Himalayas, recent decades have witnessed greater increases in maximum temperature at higher elevations, along with shortened snow cover duration and earlier spring snowmelt. These changes have reshaped the structure of the plant growing season and altered moisture supply patterns, exerting complex effects on NEP (Ghimire et al., 2025). Beyond elevation and temperature, moisture acts as a key co-regulating factor. In the Qilian Mountains, elevation has been identified as the strongest independent factor influencing the spatial variation of NEP, followed by temperature. Although precipitation plays a relatively weaker individual role, its interaction with temperature substantially enhances the spatial explanatory power of NEP (Wang, Zhao & Zhang, 2022). Long-term ecological studies conducted in the European Alps, as well as in Switzerland and Austria, have shown that plant phenology at higher elevations responds more sensitively to shortened snow cover and temperature fluctuations, while soil temperature and microbial activity exhibit greater interannual variability (Rogora et al., 2018). The effects of high solar radiation intensity and ultraviolet exposure in alpine environments should not be overlooked. Although many studies have primarily focused on plant phenology and species composition, variations in solar radiation, temperature, and soil moisture have been demonstrated to significantly influence ecosystem respiration and primary productivity in mountain ecosystems (Magnani et al., 2020).

Using interaction detectors to analyze the interactions between different driving factors and their impacts on the spatial differentiation of the vegetation NEP, the interaction between any two driving factors significantly influences the vegetation NEP more than the independent effect of any single factor does. This demonstrates a synergistic effect between pairs of factors, indicating collaborative interactions among driving factors (Chen et al., 2024). The spatiotemporal variation in the vegetation NEP in southeastern Xizang is shaped not by a single driving factor but by the combined influence of multiple factors.

This study achieved its primary objective of quantifying the spatial and temporal dynamics of vegetation NEP in southeastern Xizang and identifying its dominant driving factors based on the improved CASA model. These findings provide a better understanding of the regional carbon source–sink dynamics and the ecological responses of forest ecosystems to environmental change.

Future research could further integrate ground-based observations and flux tower data to validate the model estimates, and employ multi-source remote sensing datasets and process-based models to improve spatial accuracy and assess the long-term impacts of climate and human activities on the regional carbon balance. Although this study focused on historical NEP dynamics from 2000 to 2020, future environmental changes—such as projected warming and altered precipitation patterns in southeastern Xizang—may further affect vegetation carbon sink capacity, which warrants continued investigation.

## Conclusions

In this study, the spatial and temporal patterns and drivers of the NEP in southeastern Xizang from 2000 to 2020 were analyzed *via* the improved CASA model and remotely sensed data, and the main conclusions are as follows:

(1) The multiyear vegetation NEP in southeastern Xizang from 2000 to 2020 showed a fluctuating upward trend, and the multiyear average vegetation NEP was 519.06 gC m^−2^ a^−1^. Southeast Xizang as a whole is in a carbon sink state, the amount of carbon sequestered by vegetation is greater than that consumed by soil respiration, and the sequestration capacity of vegetation is also increasing. This provides long-term evidence for assessing the carbon sink function of forests on the Tibetan Plateau and offers data support for regional carbon budget monitoring and the achievement of national carbon neutrality goals.

(2) The spatial distribution pattern of the vegetation NEP in southeastern Xizang from 2000 to 2020 was high in the south and low in the north, high in the east and low in the west. The high-value vegetation NEP area in southeastern Xizang was located mainly in Motuo County in the south. The changes in vegetation NEP over the years were dominated by insignificant decreases, non-significant decreases, no changes, non-significant increases, and insignificant increases. It reveals the spatial heterogeneity of regional carbon sinks, providing a scientific basis for identifying key areas for conservation and carbon sink enhancement, and contributing to the optimization of spatial planning for ecological compensation and restoration.

(3) Altitude, precipitation and temperature were the main factors affecting the vegetation NEP in the study area, and the interaction of any two factors had a greater effect on the vegetation NEP than the independent effects of the individual factors did, indicating a two-factor amplification effect. Climatic factors exhibit significant synergistic effects in driving changes in carbon sinks. This finding contributes to predicting the dynamic response of NEP under climate change and provides a scientific basis for regional climate–ecosystem coupled management.

(4) By revealing the spatiotemporal patterns and driving mechanisms of vegetation NEP in southeastern Xizang, this study provides a scientific reference for assessing and dynamically regulating the carbon sink function of forest ecosystems in the region. The findings contribute to guiding the conservation and sustainable utilization of forest resources, optimizing land use and ecological restoration strategies under local hydrothermal conditions, and enhancing the carbon sequestration capacity and stability of regional ecosystems. Moreover, these insights hold important implications for ecological management in southeastern Xizang and offer valuable references for global studies on alpine forest carbon sinks and the formulation of climate change adaptation policies.

## Supplemental Information

10.7717/peerj.20572/supp-1 Supplemental Information 1 NPP

10.7717/peerj.20572/supp-2 Supplemental Information 2 NEP

## Additional Information and Declarations

### Data Availability

The following information was supplied regarding data availability: The TerraClimate Animations are available at: https://climate.northwestknowledge.net/TERRACLIMATE/index_animations.php. The Landsat Collection 2 Surface Reflectance data is available at: https://www.usgs.gov/landsat-missions/landsat-collection-2-surface-reflectance. The raw data are available in the Supplemental Files.
